# Squamous cell carcinoma over pilonidal chronic disease. A new therapeutic approach

**DOI:** 10.1016/j.ijscr.2020.04.043

**Published:** 2020-05-11

**Authors:** A. Mayol Oltra, E. Boldó Roda, R. Lozoya Albacar, V. Morillo Macias, N. Nobleja Quiles

**Affiliations:** a Department of Surgery, Consorcio Hospitalario Provincial of Castellón, Spain; b Department of Oncology Radiotherapy, Consorcio Hospitalario Provincial of Castellón, Spain

**Keywords:** Pilonidal, Sacrococcigeal, Squamous, Carcinoma, Surgery, Radiotherapy, Chemotherapy

## Abstract

**Introduction:**

Pilonidal sinus is a very common disease. Malignant transformation occurs in 0,1% of patients. We present a case of squamous cell carcinoma arised from recurrent pilonidal disease, managed by multimodal treatment.

**Presentation of case:**

We present a 70-year-old man with chronic pilonidal sinus. Inflammation had worsened in previous months and exploration revealed a large ulcerative mass which biopsy showed a squamous cell carcinoma. CT scan and MRI imaging showed tumoral invasion of the coccyx and both gluteus major muscles. Neoadjuvant radiotherapy, chemotherapy as radiosensitizer and surgery with intraoperative radiotherapy was decided in the multidisciplinary tumor committee. Post neoadjuvant therapy MRI showed partial response with a decrease of the mass but persistence of the coccyx infiltration.

Surgery consisted in en-bloc resection of the tumor with presacral tissues, coccyx and partial gluteal resection. Intraoperative radiotherapy was administered over the sacrum and in the bed of the coccyx resection. One week later, reconstructive surgery was practiced using a latissimus dorsi free flap, advancement of gluteal flaps and skin graft. Histological examination showed no residual tumor. The patient is currently asymptomatic and he has a satisfactory quality of life.

**Discussion:**

Although squamous cell carcinoma is rare, it must be suspected in patients with recurrent pilonidal disease. Diagnosis is done by histological examination of biopsies. This type of tumors have a high local recurrence rate.

**Conclusion:**

We propose a multimodal treatment that includes neoadjuvant radiotherapy and chemotherapy as radiosensitizer and surgery plus intraoperative radiotherapy with the aim to decrease local recurrence rate.

## Highlights

• Squamous cell carcinoma can appear in chronic pilonidal disease.
• En bloc surgery is the main treatment.
• Intraoperative radiotherapy could decrease the recurrence rate.

## 1 Introduction

Pilonidal sinus is a common disease that may affect up to 5% of the general population [[1], [2], [3], [4]]. The most frequent complication is infection [[1], [2], [3], [4]]. Malignant degeneration occurs in 0,1% of patients, only reported in recurrent pilonidal disease [1,5,6]. The most frequent tumor type is squamous cell carcinoma [[7], [8], [9], [10]]. Our objective is to report a case of a patient with a squamous cell carcinoma secondary to chronic pilonidal disease managed with multimodal treatment that obtained total pathological response. It included chemotherapy and external radiotherapy with neoadjuvant intention followed by en-bloc resection with intraoperative radiotherapy and reconstructive surgery.

The work has been reported in line with the SCARE criteria [11].

## 2 Presentation of case

**Fig. 1:**
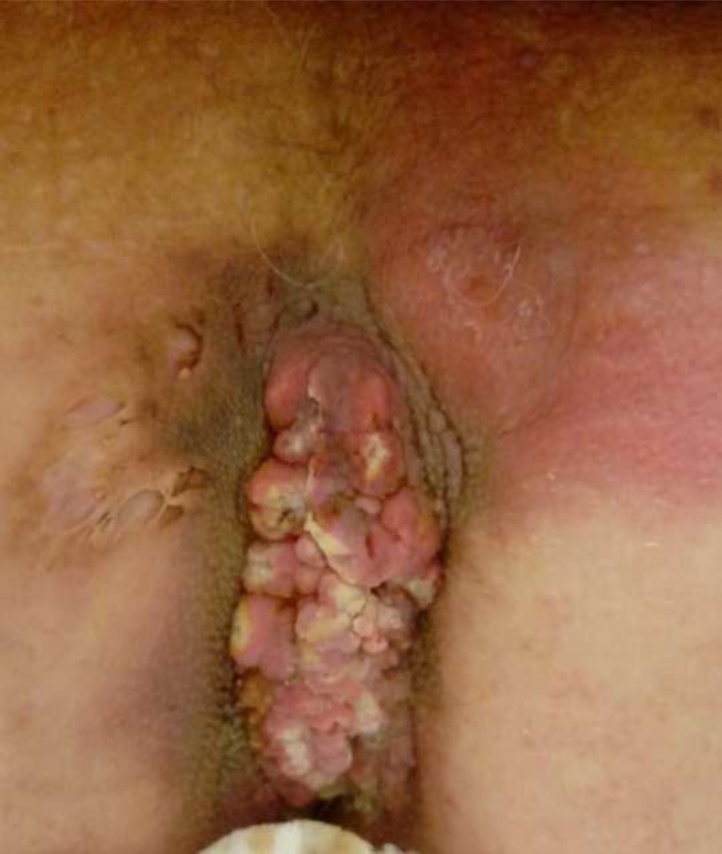
Ulcerated mass in the sacrococcigeal area.

A 70-year-old male with pilonidal disease of 20 years of evolution was referred to our department. Inflamation had worsened in the previous months. Clinical examination revealed an ulcerated mass in the sacrococcigeal area (Fig. 1) and unilateral enlarged inguinal lymph nodes. Ano-rectal exploration was negative. Incisional biopsy of the sacrococcigeal mass showed well differentiated squamous cell carcinoma and FNA of the lymph nodes was negative for malignant cells.

**Fig. 2:**
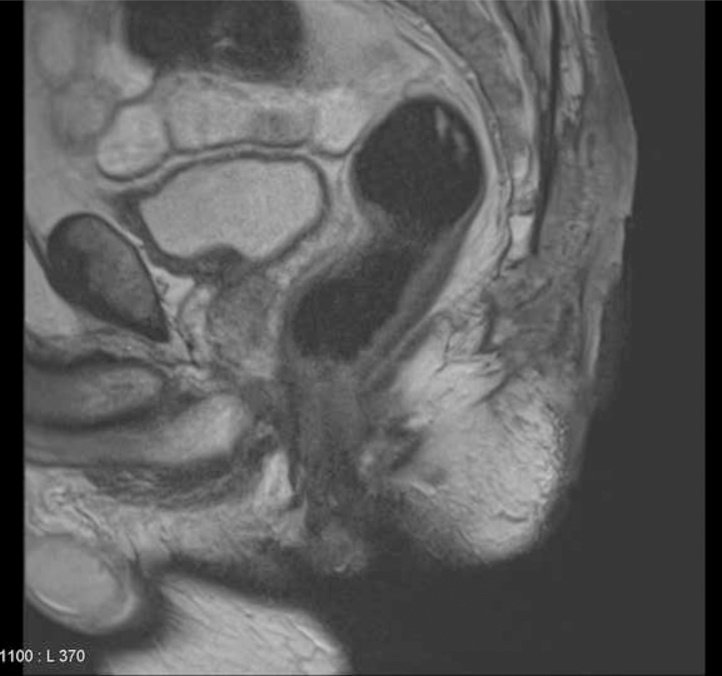
MRI imaging before neoadjuvant treatment (sagital view).

Magnetic resonance imaging (MRI) showed a 107 × 45 × 37 mm mass infiltrating both gluteus major muscles and the last coccigeous vertebrae (Fig. 2). Chest, abdominal and pelvic CT scan showed no distant metastasis.

Neoadjuvant radiotherapy, chemotherapy as radiosensitizer and surgery with intraoperative radiotherapy was decided in the multidisciplinary tumor committee. The radiotherapy scheme was 59,4 Gy over tumor and lymph nodes larger than 3 cm (1,98 Gy per session), 50,4 Gy over lymph nodes smaller than 3 cm, (1,68 Gy per session) and 45 Gy over pelvis (1,5 Gy per session) [12]. The chemotherapy used was 825 mg/m^2^ of capecitabin orally.

**Fig. 3:**
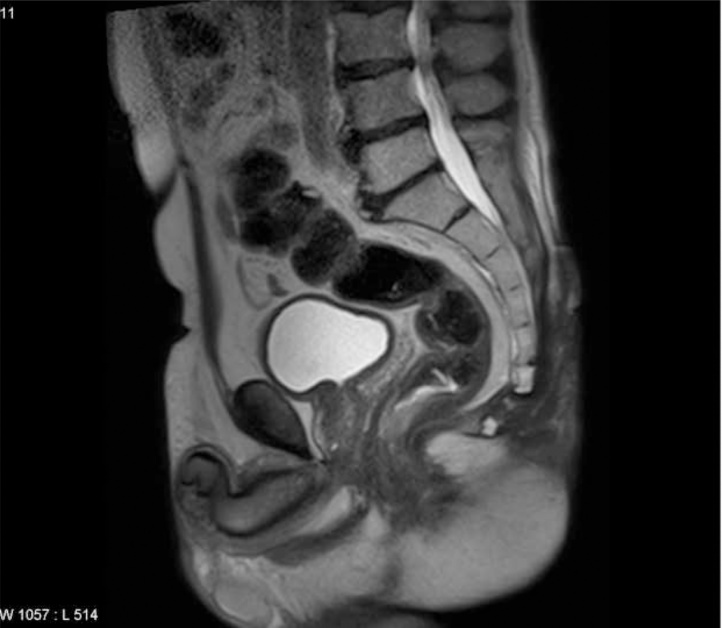
MRI imaging after neoadjuvant treatment(sagital view).

Post neoadjuvant therapy MRI showed partial response with a decrease of the mass but persistence of the coccyx infiltration (Fig. 3).

**Fig. 4:**
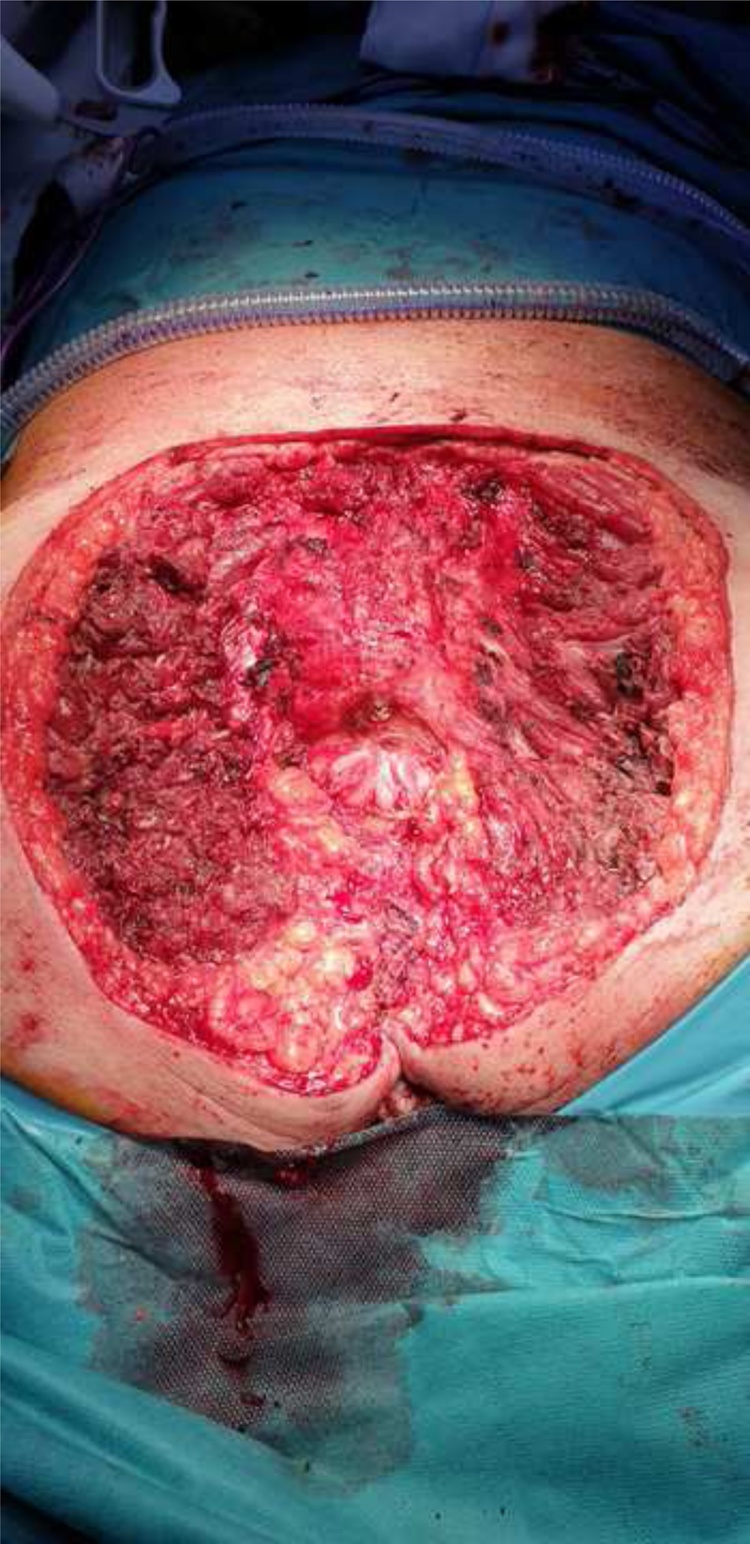
Surgical result after en-bloc resection.

Surgery consisted in en-bloc resection of the tumor with presacral tissues, coccyx and partial gluteal resection (Fig. 4). Intraoperative radiotherapy (10 Gy, 90% isodose 9mEv energy) over the sacrum and in the bed of the coccyx resection was administered. Reconstructive surgery using a latissimus dorsi free flap, advancement of gluteal flaps and skin graft was practiced by plastic surgeons one week later. No postoperative complications were registered.

**Fig. 5:**
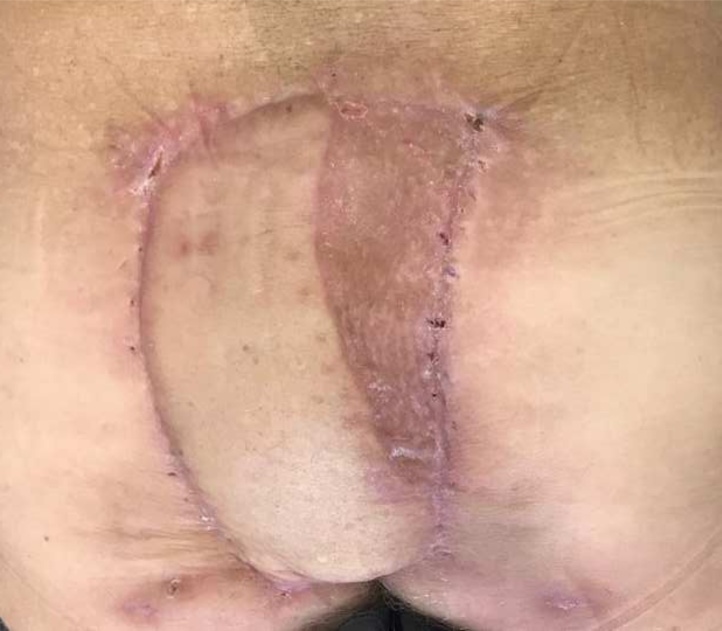
Cosmetic result after reconstructive surgery (one month after surgery).

Path report showed inflammatory infiltration without residual tumor. Adjuvant treatment was discarded and the patient followed up every 3 months. Cosmetic results are shown in Fig. 5. The patient is disease free at the moment of this report (12 months).

## 3 Discussion

We report a case of rare tumor arising from a common disease that must be suspected and can be treated efficiently by multimodal therapy.

The first case of malignant transformation of pilonidal disease was reported by Wolff et al. in 1900 [1,13,14]. The most frequent tumor type is squamous cell carcinoma [[7], [8], [9], [10]]. Other tumor types reported are basal cell carcinoma, mixed squamous and basal carcinoma and adenocarcinoma and unspecified tumor [1,15].

Pathogenesis of malignant degeneration is similar to that associated with other chronic cutaneous disorders. It is caused by the release of free oxygen radicals inducing genetic damage and neoplastic transformation [7,8,10]. Likewise, the normal repair DNA mechanism is impaired in chronic inflammation and predisposes to malignancy [7,10]. Before malignant transformation, long lasting recurrent pilonidal disease has always been reported [1,7]. Thus, when central ulceration or a mass appears in this clinical setting, biopsy is mandatory [1,7,16].

Preoperative evaluation should include exploration of the inguinal lymph nodes and digital rectal exam [1,7,13,16,17]. Recto-sigmoidoscopy should be done if there is suspicion of involvement of the rectum [7,10,16,17]. Image examinations with CT scan and MRI reveal local and lymph node infiltration of the tumor and help in treatment planification. A chest, abdomen and pelvic CT scan must be done to diagnose distant metastases [[16], [17], [18], [19]].

In the presence of enlarged inguinal lymph nodes, FNA or core biopsy must be taken. At the time of the diagnosis 14% of the patients have nodal metastasis. Inguinal lymph nodes metastasis are associated with poor prognosis, with a median survival of 2 years [20,21]. Our patient´s MRI showed infiltration of the coccyx and gluteal muscles. Eight per cent of the patients have bone invasion at diagnosis [1,20].

According to literature review, the treatment of choice is en-bloc resection including at least presacral fascia, subcutaneous fat tissue, muscle and often bone resection, like in our patient. If the rectum or the anus are infiltrated, an abdominoperineal resection (with sacrum resection) must be performed [1,[5], [6], [7],[16], [17], [18]]. Prophylactic lymphadenectomy has not been recommended [7,18].

The resulting defect can be covered using skin grafts, local flaps or free musculo-cutaneous flaps [1,5,6,16]. In our case, a combination of those techniques. was chosen, due to large defect covering needs.

Local recurrence rate is 40–50% and often occurs during the first year after surgery [1,7,17]. Adjuvant radiotherapy associated to free surgical margins decreases local recurrence to 30% [7,10,16] The role of adjuvant chemotherapy is unclear. It can be considered in high-risk lesions (lymphovascular/perineural invasion, resected tumor positive margins), in combination with surgery and radiotherapy [7,16,19]. To the best of our knowledge, we used a completely new approach: radiotherapy as neoadjuvant treatment and chemotherapy with capecitabin as radiosensitizer. The aim of this approach was decrease tumor size and thus the need for more aggressive surgery. Besides, with this scheme pathological analysis showed complete response. In addition, intraoperative radiotherapy was administered on the high-risk surface (sacrum and bed of the coccyx resection) with the purpose of decreasing local recurrence risk in the area with a greater danger of recurrence. It also decrease the radiation dose on the other neighboring organs. Given the complete pathological response and the absence of high risk factors, it was decided to not administer adjuvant treatment.

Postoperative follow-up includes: clinical examinations of the sacrococcygeal and inguinal area, inguinal ultrasound if there is evidence of suspicious inguinal lymph nodes, and CT of abdomen to rule out local recurrence or distant metastasis, every 3 months for the first 2 years, every 6 months for the next 3 years and once every year after that [16].

The 5-year survival rate is almost 55–61%. Metastasis appears in 14% of patients and is usually fatal [7,16].

## 4 Conclusions

In conclusion, squamous cell carcinoma is a rare tumor that occurs in 0,1% in patients with chronic pilonidal disease. Preoperative evaluation includes clinical examination of the area and inguinal lymph nodes, complementary explorations like biopsy and pelvic MRI. The chest, abdomen and pelvic CT scan is recommended to rule out distant metastasis. A multidisciplinary approach is recommended. Surgery is the main curative treatment. Radiotherapy and chemotherapy as neoadjuvant treatment can be considered in selected cases like large tumors. In addition, intraoperative radiotherapy may help in reducing the local recurrence rate without affecting the neighboring organs.

## Declaration of Competing Interest

None.

## Funding

Funds from the Foundation of the Provincial Hospital of Castellon. It no has any involvement.

## Ethical approval

Ethics approval and consent to participate: not applicable.

## Consent

Written informed consent was obtained from the patient for publication of this case report and accompanying images.

## Registration of research studies

NA.

## Guarantor

Araceli MAyol Oltra.

## Provenance and peer review

Editorially reviewed, not externally peer-reviewed.

## CRediT authorship contribution statement

**A. Mayol Oltra:** Conceptualization, Investigation, Resources, Writing - original draft, Writing - review & editing. **E. Boldó Roda:** Writing - review & editing, Supervision. **R. Lozoya Albacar:** Supervision. **V. Morillo Macias:** Writing - review & editing, Supervision. **N. Nobleja Quiles:** Supervision.
